# Cryptic speciation in the Acari: a function of species lifestyles or our ability to separate species?

**DOI:** 10.1007/s10493-015-9954-8

**Published:** 2015-07-26

**Authors:** Anna Skoracka, Sara Magalhães, Brian G. Rector, Lechosław Kuczyński

**Affiliations:** Department of Animal Taxonomy and Ecology, Institute of Environmental Biology, Faculty of Biology, Adam Mickiewicz University, Poznan, Poland; Centre for Ecology, Evolution and Environmental Changes (cE3c), Faculty of Science, University of Lisbon, Lisbon, Portugal; USDA-ARS, Great Basin Rangelands Research Unit, Reno, NV USA; Department of Avian Biology and Ecology, Institute of Environmental Biology, Faculty of Biology, Adam Mickiewicz University, Poznan, Poland

**Keywords:** Biodiversity, Cryptic species, Hidden diversity, Integrative approach, Mites and ticks, Molecular systematics, Taxonomy

## Abstract

There are approximately 55,000 described Acari species, accounting for almost half of all known Arachnida species, but total estimated Acari diversity is reckoned to be far greater. One important source of currently hidden Acari diversity is cryptic speciation, which poses challenges to taxonomists documenting biodiversity assessment as well as to researchers in medicine and agriculture. In this review, we revisit the subject of biodiversity in the Acari and investigate what is currently known about cryptic species within this group. Based on a thorough literature search, we show that the probability of occurrence of cryptic species is mainly related to the number of attempts made to detect them. The use of, both, DNA tools and bioassays significantly increased the probability of cryptic species detection. We did not confirm the generally-accepted idea that species lifestyle (i.e. free-living vs. symbiotic) affects the number of cryptic species. To increase detection of cryptic lineages and to understand the processes leading to cryptic speciation in Acari, integrative approaches including multivariate morphometrics, molecular tools, crossing, ecological assays, intensive sampling, and experimental evolution are recommended. We conclude that there is a demonstrable need for future investigations focusing on potentially hidden mite and tick species and addressing evolutionary mechanisms behind cryptic speciation within Acari.

## Introduction

The evolution of the diversity of life on Earth was called “the mystery of mysteries” by Darwin (1859). The extraordinary diversity of species of mites and ticks (Arachnida: Acari), comprising a vast array of morphological, biological and ecological variation, has inspired acarologists for decades and compels us to understand the evolutionary and ecological processes underlying the origin and proliferation of such diversity. Approximately 55,000 Acari species (including Acariformes and Parasitiformes) have been described, accounting for almost half of all known Arachnida species, and 3.5 % of all Animalia species discovered so far (Zhang 2011). Moreover, estimates of total mite and tick diversity are far greater, reaching 500,000–1,000,000 species (Zhang 2011; Walter and Proctor 2013). One important source of currently hidden Acari diversity is cryptic speciation, i.e. the development of reproductive barriers within an ancestral species that leads to new reproductively isolated species that are virtually identical in their morphology (e.g. Bickford et al. 2007). This poses a challenge to taxonomists, who traditionally distinguish between species based on morphological characters. Such cryptic species are believed to be responsible for gross underestimates of Acari biodiversity.

Cryptic species are commonly defined as species that are difficult to distinguish using traditional morphology-based taxonomic methods (Knowlton 1993), or species classified as a single nominal species because they are at least apparently morphologically indistinguishable (Bickford et al. 2007). Some studies explain this phenomenon as a result of recent speciation, after which detectable morphological traits have yet to appear; such cryptic species are evolutionarily young forms that are more similar genetically than more typical, readily distinguishable species (Saez and Lozano 2005; Cooke et al. 2012). There are also empirical examples of cryptic species that do not represent the initial stage of speciation, suggesting the possibility of cryptic speciation in extreme environments or retention of highly conserved morphology due to stabilizing selection in homogenous habitats (Colborn et al. 2001; Lefebure et al. 2006). However, the biological nature of many cryptic species has been questioned by some authors because of the inadequacy of morphological methods or insufficient thoroughness in their application during species description (Knowlton 1993; Lajus et al. 2015). In fact, the application of new technologies (e.g. DNA methods, advanced microscopy) allowed detection of morphological or ecological differences between species previously considered to be cryptic (e.g. Hebert et al. 2004; Padial and de la Riva 2009; Cheng et al. 2011). Regardless of the biological or methodological definition of cryptic species, their presence concerns specialists in a broad range of scientific and applied areas.

A thorough understanding of the extent of cryptic diversity within any given taxonomic group is essential not only to assess its overall diversity but also to recognize the complexity of its ecological interactions and evolutionary histories. Given the great economic and medical importance of many mite and tick species, whether as parasites, crop pests, pathogen vectors, or biological control agents (e.g. Navia et al. 2013a; Walter and Proctor 2013), their misidentification may have serious negative consequences for human activities (e.g. Anderson and Trueman 2000; Bernasconi et al. 2002; Arthur et al. 2011; Beati et al. 2013; Matsuda et al. 2013; Miller et al. 2013; Navia et al. 2013b; Skoracka et al. 2013; Burger et al. 2014). Indeed, neglected cryptic diversity may hamper the development of technologies and management tools in medicine, agriculture and other important fields, due to the inability to link cryptic species to their unique epidemiological, pathogenic or host-specific traits (Armstrong and Ball 2005; Pringle et al. 2005; Bickford et al. 2007).

### Why are cryptic species expected in the Acari?

Mites occupy almost every habitat on Earth, with the exception of the water column of the open ocean (Walter and Proctor 2013), but including extreme habitats such as the Antarctic and hypersaline lakes (Stevens and Hogg 2006; Moreno et al. 2008). This ubiquity increases the likelihood that a large number of mite species remains undetected. In addition, environmental heterogeneity on smaller scales results in more available niches for microscopic animals such as mites compared to larger organisms, and may contribute to the development of more species per unit area.

Regardless of the terrestrial or aquatic habitats that Acari occupy, many species have evolved under facultative or obligatory associations with other organisms (i.e. hosts) that function as their permanent or temporary habitats (Walter and Proctor 2013). Such associations [herein symbioses, sensu Wilkinson (2001)] are often highly specific. It has been suggested that a symbiotic lifestyle may drive speciation (de Meeûs 2000). Many studies of symbiotic invertebrates have suggested that speciation via host specialization was responsible for the development of cryptic species (e.g. Stireman et al. 2005, 2010; Steinauer et al. 2007; Tsang et al. 2009). Walter and Proctor (2013) postulated that the species richness of symbiotic mites will be largely a function of the extant diversity of their hosts. However, to date, the relationship between the lifestyle and the abundance of cryptic species in Acari has been not rigorously tested.

Another reason to expect that cryptic species are common in Acari is the phenomenon of morphological stasis, i.e. diversification that leads to genetic isolation in the absence of apparent morphological differentiation, which has been revealed in many taxa (e.g. Hansen et al. 2001; Lee and Frost 2002; Pfenninger and Schwenk 2007; Halt et al. 2009; Spencer et al. 2009; Jesse et al. 2010). Given that many mite species are virtually blind, the reproductive behaviour of mites often involves non-visual signals such as tactile stimulation or chemical communication (e.g. Rock et al. 1976; Evans 1992; Michalska et al. 2010). Indeed, mate recognition between morphologically cryptic species is often based on cues other than morphological traits (e.g. Henry and Wells 2010; Funk et al. 2011). Moreover, several mite species inhabit marine habitats (e.g. Bartsch 2004; Pfingstl 2013; Walter and Proctor 2013), where speciation processes may often be coupled to chemical recognition (Knowlton 1993). As such, the possibility of morphologically static cladogenesis in the Acari should not be ignored.

Finally, due to their minute size and simplified body form, species discrimination within many groups of mites (e.g. Eriophyoidea, Demodicidae, Oribatida) is severely constrained when reliant on the traditional morphological techniques that are still prevalent in mite taxonomy today (e.g. de Lillo et al. 2010; Zhao et al. 2013). This handicap in species identification can be explained in part by the inherent limitations in the state of the art of commonly available microscopy and the relatively small amount of morphological details it can reveal. Given that in systematics the most conspicuous and the most utilized traits are morphological, this also suggests the undetected presence of cryptic species, particularly in taxa with minute organisms such as the Acari.

Simply put, cryptic species may be much more prevalent in the Acari than commonly thought, due to their ecology or to human perception bias.

### The objective

Recently, Magalhães et al. (2007) reviewed the occurrence of host race formation in parasitic mites and ticks. Since then many new data have become available that suggest that many of what were previously considered host races may be in fact cryptic species (e.g. Skoracka and Dabert 2010). In the present paper we revisit the subject of biodiversity in the Acari and investigate what is currently known about cryptic species within this group, including all Acari taxa. Specifically, we ask whether the level of cryptic diversity detected within the Acari is related to:

1. The organism lifestyle (free-living vs. symbiotic),
2. The relative amount of research effort devoted to each Acari group,
3. The methods applied by researchers.

## Methodology

### Literature review and data pre-processing

Our aim was to determine the proportion of cryptic species present in the Acari relative to the total number of described species in a given taxon (due to data sparseness we chose the superfamily as the basic unit for our analysis). To identify studies of cryptic species within the Acari, we selected articles published from 1960 to July 2014 by searching the SCOPUS database http://www.scopus.com) using the following query: [TITLE-ABS-KEY (“cryptic species” OR “cryptic speciation” OR “sibling species” OR “species complex”)] AND [TITLE-ABS-KEY (acari OR mites OR mite)]. Simply put, all papers were searched within the title, abstract or keywords for: (i) at least one occurrence of the following terms:“cryptic species”, “cryptic speciation”, “sibling species”, “species complex”; and (ii) at least one occurrence of the terms: “Acari”, “mites”, “mite”. The two search criteria were combined and only papers fulfilling both were selected. Thereafter, only research articles (either published or in press) were taken into account. According to the query used, 149 articles were found on the SCOPUS database. The abstracts of all articles were screened to assess the aims of each study and the group of organisms studied. Articles that focused on organisms other than the Acari or topics other than cryptic species were excluded from further analysis. In total, 108 papers were selected, from which we noted: (i) the study organism, including its superfamily classification (ii) the methods applied by the authors for species identification, i.e. morphological or molecular characters, bioassay (ecological assay or crossing), (iii) whether the study organism was involved in any symbiotic relationship in any part of its life cycle (e.g. parasitism, phoresy); (iv) whether the study revealed the occurrence of cryptic species or a complex of cryptic species within the study organism.

We then linked this publication data with a database containing information on the number of species within taxonomic groups. To obtain the total number of species, we used Zhang (2011), and specifically the following chapters: Suborder Oribatida (Schatz et al. 2011), Suborder Parasitiformes (Beaulieu et al. 2011), and Order Trombidiformes (Zhang et al. 2011), from which we noted the number of all species described within the each superfamily.

The final database contained the following information: (i) the number of all species described within each superfamily, (ii) the number of cryptic species or species complexes found within each superfamily, (iii) symbiotic relationships of any representatives within the superfamily, (iv) the methods used by authors for species identification.

### Data analysis

To check how the probability of cryptic species occurrence within each superfamily is related to the methods applied by researchers or the lifestyle of the Acari under study, we used generalized linear models (GLM). The response in our binomial model was the probability of success, i.e. the number of cryptic species detected to the number of all species described so far within a given superfamily. Predictors coding for the research effort were: (i) the use of DNA-based methods (0 = No, 1 = Yes); (ii) the number of taxa analyzed for the presence of cryptic species; and (iii) the use of bioassays (0 = No, 1 = Yes). All two-way interactions between above mentioned variables were included in the model, allowing for testing the potential acceleration in cryptic species detection when using an integrated approach. Additionally, to test whether the lifestyle affected the probability of cryptic speciation, we included a variable coding for whether any representatives within the superfamily were involved in such association (0 = No, 1 = Yes). Statistical analyses were performed using R 3.1.2 (R Development Core Team 2014).

## Results from the literature review

All described Acari species are classified into 142 superfamilies (Zhang 2011), whereas cryptic species have only been found in 24 superfamilies (ca. 17 %). The largest numbers of known cryptic species were within the Eriophyoidea and Dermanyssoidea (ca. 40 in each group). The suborders with no records of studies addressing cryptic speciation to date included: Endeostigmata, Sphaerolichida, Opilioacarida, Holothyrida, Sejida, and Trigynaspida.

### Statistical analysis

#### Acari lifestyle and cryptic diversity

**Fig. 1:**
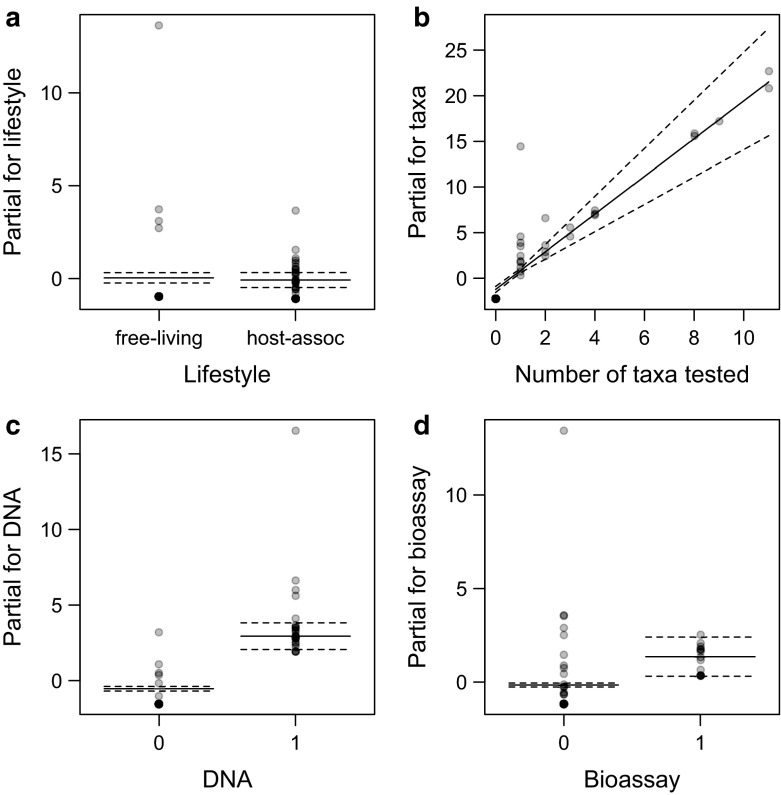
Results of the generalized linear model presenting the effect of the life style (**a**), the number of taxa verified for the existence of cryptic species (**b**), if DNA (**c**) or bioassays (**d**) were used (0 = No, 1 = Yes) of the studied Acari on the probability of cryptic species occurrence within a superfamily. Values on *vertical axes* are partial residuals. *Solid lines* are estimates and *dashed lines* are standard errors around them

Lifestyle (symbiotic vs. free-living) had no effect on the number of cryptic species detected within the Acari (Table 1; Fig. 1a). Thus, strong host relationships may promote genetic differentiation and lead to host race formation (Magalhães et al. 2007) but this does not necessarily lead to the evolution of reproductive isolation. Alternatively, host-race formation may often induce speciation but no more so than abiotic and other host-independent environmental factors. For example, Edwards et al. (1999) showed that speciation via host specialization among unionicolid water mites can be influenced by the interplay of differences in the geographic distributions of available hosts and geographic variation in host utilization. Niche differentiation due to environmental conditions was suggested to drive *Amblyomma cajannense* tick diversity rather than host associations (Beati et al. 2013). In the bee-associated mite genus *Chaetodactylus*, geographic isolation and temporal isolation (i.e. bee activity in different seasons) have been suggested as factors influencing the separation of two cryptic species (Klimov and OConnor 2004). Notwithstanding, our analysis concludes that Acari species with a symbiotic lifestyle are no more subject to cryptic speciation than those with a free-living lifestyle.

**Table 1:** Parameter values (on the logit scale) of the model relating the proportion of cryptic species detected within each superfamily with their lifestyle (0: free-living, 1: symbiotic), the number of taxa analyzed for the presence of cryptic species and the research methods used (the use of DNA-based methods 0 = No, 1 = Yes; the use of bioassays 0 = No, 1 = Yes)

Parameter	Estimate	SE	z value	*p*
Intercept	−8.50	0.46	−18.52	<0.0001
Lifestyle	−0.12	0.34	−0.34	0.73
No. of taxa analyzed	2.06	0.28	7.29	<0.0001
DNA	3.48	0.52	6.71	<0.0001
Bioassay	1.52	0.58	2.63	0.0085
Taxa:DNA	−2.10	0.29	−7.16	<0.0001
Taxa:bioassay	0.06	0.10	0.61	0.54
DNA:bioassay	−1.14	0.66	−1.72	0.086

All two-way interactions between variables coding the research effort (no. of taxa analyzed, the use of DNA, the use of bioassays) were included in the model, allowing for testing the potential acceleration in cryptic species detection when using an integrated approach

#### Detection of cryptic species

The probability of cryptic species occurrence within each superfamily was found to be mostly related to the number of attempts made to detect them (Table 1; Fig. 1b). The use of either DNA analysis or bioassays significantly increased the probability of cryptic species detection (with DNA being several times more effective than bioassays; Table 1; Fig. 1c, d). There was a significant interaction between the number of taxa checked and the use of DNA analysis (Table 1).

### Methods employed to detect hidden mite diversity

#### DNA-based methods

The advent of rapid DNA sequencing technologies has revealed that cryptic diversity is unexpectedly common in virtually all taxonomic groups (e.g., Hansen et al. 2001; Pfenninger and Schwenk 2007; Halt et al. 2009; Spencer et al. 2009; Jesse et al. 2010; Cardeñosa et al. 2014; Nantarat et al. 2014) and the Acari are no exception. Approximately 63 % of studies focusing on Acari species complexes have used molecular methods. Among these, ca. 40 % employed a single DNA marker, the remaining 60 % applied from two to five markers. The most frequently used marker was the mitochondrial cytochrome *c* oxidase subunit I (mtCO1; 60 % of studies), which is widely adopted as a DNA barcode (Hebert et al. 2003). Among nuclear DNA markers the most frequently used in Acari studies were internal transcribed spacer [ITS] regions of the ribosomal cistron (ca. 37 %) and nucleotide sequences of the large subunit (28S) of the rDNA gene (23.5 %). More rarely other mitochondrial (viz. 16S rDNA, 12S rDNA, cyt b, COII, d-loop) and nuclear (viz. adenine nucleotide translocator [ANT], elongation factor 1 alpha [ef-1α], the genes encoding heat shock protein 82 [hsp82], lysosyme, tropomyosin intron) sequences; Restriction Fragments Length Polymorphism (RFLP), Amplified Fragment Length Polymorphism (AFLP) and elecrophoretic analyses were used. Two studies used complete mitochondrial genome sequences to detect cryptic species (Liu et al. 2013; Burger et al. 2014).

#### Morphological methods

A wide range of morphological methods have been employed alone or in combination with other tools to assess cryptic diversity in Acari. In addition to traditional taxonomy and simple statistics, occasionally supplemented by scanning electron microscopy or DNA data (e.g. Walter and Campbell 2003; Dabert et al. 2008; Skoracka 2009; Navia et al. 2013b), a broad spectrum of morphometric multivariate analyses have advanced our knowledge of cryptic mite diversity. These include: (i) logistic regression; (ii) principal component analysis [PCA]; (iii) canonical correlation analysis; (iv) discriminant function analysis; (v) multidimensional scaling; (vi) hierarchical clustering; (vii) classification trees; and (viii) phylogenetic reconstruction (e.g. Deunff et al. 2004; Klimov et al. 2004, 2006; Klimov and OConnor 2004; Roy et al. 2009; Schäffer et al. 2010; Heethoff et al. 2011; Skoracka et al. 2012, 2014). Two studies applied geometric morphometrics, i.e. a landmark-based approach that allows a geometric representation of forms (Jagersbacher-Baumann 2014; Vidovic et al. 2014), although in one of these studies (Jagersbacher-Baumann 2014) traditional morphometrics performed better than geometric morphometrics.

#### Ecological assays and crosses

Ecological assays were employed in 19 (17.5 %), and crossing experiments in 17 (16.6 %) of all studies. The small number of studies that have analyzed crosses between suspected cryptic species can be attributed to the great technical difficulty inherent to such experiments in many Acari due to their small size, as well as the inability to conduct such trials in strictly parthenogenetic species. However, the direct measurement of reproductive compatibility is commonly considered to be the best tool to distinguish between species. In some studies, this approach has supported results derived from other methods (DNA or morphological) indicating the existence of valid species (e.g. Klimov et al. 2004; Tixier et al. 2006; Skoracka 2008; Famah-Sourassou et al. 2012). In other studies, crossing experiments led to rejection of hypotheses regarding the existence of cryptic species (e.g. Lysyk and Scoles 2008; Navia et al. 2014), emphasizing the power of this technique.

Cryptic species, while lacking conspicuous morphological differences, may differ in physiological, behavioural or ecological traits (e.g. Calcagno et al. 2010; Henry and Wells 2010). Ecological and chemical assays that have been employed to study Acari species complexes showed that cryptic species may differ in their host ranges (e.g. Evans et al. 2008; Skoracka 2009; Skoracka et al. 2013; Lewandowski et al. 2014), behaviour (Skoracka et al. 2007), demographic parameters (Skoracka and Kuczyński 2006a, b), pathogen or endosymbiont infestation (Bernasconi et al. 2002), infestation parameters (Skoracka and Kuczyński 2012; Lareschi et al. 2013), climate preference (Hill et al. 2012), oil gland secretion profiles (Heethoff et al. 2011), ability to transmit viruses (Schiffer et al. 2009), or tolerance to pesticides (Umina and Hoffman 1999; Robinson and Hoffman 2000). Ecological assays have rarely been used as the sole method of discrimination between species (e.g. Estrada-Peña et al. 1993); typically complementing molecular, morphological or crossing techniques or presumed ecological differences within already known species complexes.

#### Integrative approaches

Above we characterized four general methods used in studies of mite species complexes (viz. molecular and/or morphological characters, crosses, and ecological analyses), including attempts at combining these different approaches. Such integrative investigations are probably more powerful strategies for characterizing mite and tick diversity, as compared to single method approaches (Schlick-Steiner et al. 2010). Among studies on cryptic species in Acari, only 32.4 % integrated multiple techniques (two methods, ca. 26 %; three methods, 6.4 %). The improved rate of detection of cryptic species when using DNA sequencing (e.g. Pfenninger and Schwenk 2007) suggests that molecular data should be incorporated with alpha taxonomy whenever possible. Morphological and ecological studies should also be included to complement the molecular data. Ideally, crossing experiments would complete the analyses. An integrated approach can also provide valuable information to help identify the causes and drivers of diversification (e.g. Pfingstl et al. 2014) as well as providing more ecological and taxonomic information about the study species. For example, members of the eriophyoid species complex *Abacarus hystrix* have been distinguished as separate species on the basis of mtCO1 and 28S rDNA sequences and tests of incompatibility using crosses (Skoracka 2008; Skoracka and Dabert 2010). Ecological assays showed that these cryptic species differed in demographic parameters, behaviour, and host range (Skoracka and Kuczyński 2006a, b; Skoracka et al. 2007). Additionally, scanning electron microscopy study disclosed that they differ in the length and shape of the wax cover produced by the dorsal ridges (Skoracka 2009), suggesting possible physiological difference between these cryptic species. Genetic differentiation between several cryptic Acari species, inferred from DNA barcode data or cross-breeding experiments, has been subsequently supported by morphological re-analysis in numerous studies (e.g. Baker and Schwarz 1997; Anderson and Trueman 2000; Tixier et al. 2006; Dabert et al. 2008; Famah-Sourassou et al. 2012; Navia et al. 2013b; Pfingstl et al. 2014). These results allow for the evaluation of taxonomic characters in a phylogenetic context and suggest new distinguishing characters that can serve as diagnostic tools, often leading to the description of new species. Moreover, the application of several methods may also prevent inaccurate conclusions about the existence of separate species suggested by single method approaches (e.g. deep mitochondrial DNA divergence between lineages; Leo et al. 2010).

## Major challenges

### Intensive sampling to detect cryptic species: wheat curl mite as a case study

An issue of great significance that we did not analyse or discuss above (as it was not emphasized in the reviewed literature), is the impact of sampling strategy on detection of cryptic species. In order to accurately estimate both basic ecological parameters (infestation, population density, etc.) and the level of cryptic diversity, the design of a sampling scheme is critical. Spatial replication should be assured by covering many localities and sampling many habitat patches (hosts, soil samples, etc.) per locality. Localities should be selected randomly and their geographic positions should be recorded, together with extensive habitat data. Ideally, temporal replication should be done, to ensure the detection of species that are only occasionally present and to allow robust statistical estimation of detectability.

**Fig. 2:**
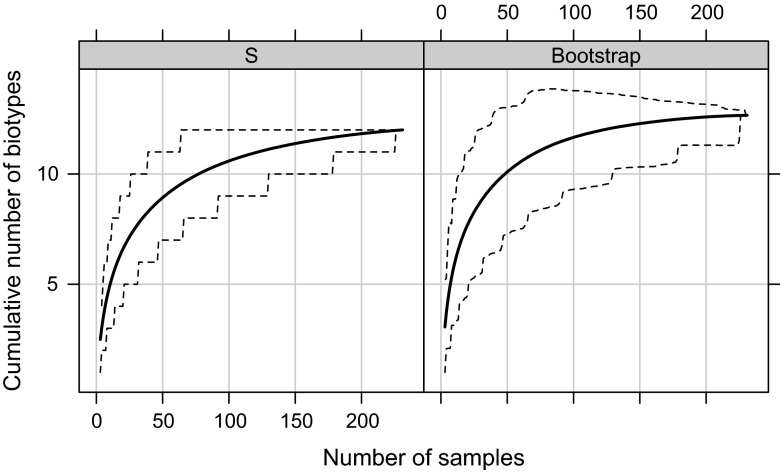
Accumulation curves and their 95 % confidence intervals for *Aceria tosichella* cryptic species resulting from an intensive sampling scheme. As the sampling effort increased, the total number of recorded genetic lineages rose and reached an asymptote that corresponds to an estimated size of the total lineage pool (including those lineages yet unknown). It was recently found (Chiu et al. 2014) that this type of estimate gives only the lower limit of the richness parameter. Thus, it is likely that the “true” number of genetic lineages within the *Aceria tosichella* complex (provided that sampling is restricted to the same geographic region, range of habitats and host species) will be higher. The panels represent different nonparametric methods of estimation of accumulation curves: S—no. of biotypes, Bootstrap—bootstrap estimator, described in Smith and van Belle (1984). Calculations were made in R using the function “specpool” from the package “vegan” (Oksanen et al. 2013)

Proper sampling strategy enables detection of species with limited ranges or those that are locally rare. Less intensive sampling (few localities or few samples per locality) may thus underestimate cryptic diversity, detecting only the most widespread and common genotypes (e.g. Funk et al. 2011). Intensive sampling schemes have rarely been utilized in studies of the Acari (e.g. Hill et al. 2012). In many groups, e.g. in eriophyoid mites, many informative data have been collected serendipitously (rev. in Skoracka et al. 2010). For example, in the course of a study on host specificity of different cryptic species within the wheat curl mite (WCM, *Aceria tosichella*) complex, seven lineages were detected based on fortuitously collected field data (Skoracka et al. 2013). In a separate study on spatial distribution of WCM cryptic species (L. Kuczyński and A. Skoracka, unpublished data), we have applied intensive, random and quantitative sampling across the total area of Poland (>300,000 km^2^), and discovered twice as many cryptic species as were previously identified (Fig. 2). Insufficient sampling may thus be an important reason that cryptic diversity remains poorly characterized within the Acari.

### The role of ecology in driving cryptic speciation

Among studies that detected cryptic species in Acari, some authors suggested that adaptation to different ecological niches accompanied the evolution of specific taxa, e.g. the adaptation of *Tectocepheus* oribatid mites to different layers in the soil (Laumann et al. 2007) or that of *Stratiolaelaps* laelapid mites to rainforests with different temperatures (Walter and Campbell 2003). Adaptation to different environmental conditions has also been shown for two cryptic oribatid species, each associated with different ecological niches within marine zones in Bermuda (mangrove forest vs. rocky or muddy substrate) (Pfingstl et al. 2014), although it is not clear whether ecological factors influenced their differentiation. Pfingstl et al. (2014) suggested that these two species diverged five to six million years ago, long before the emergence of the Bermuda landmass, and their present distribution on this archipelago is ascribed to later colonization events. Thus, it is not obvious whether their differentiation is the effect of ecological adaptation, geographic isolation or both.

However compelling the hypothesis that environmental switches promote speciation may be difficult to test. Indeed, assessing whether speciation preceded or followed a habitat switch is in general impossible in natural systems. Experimental evolution is a powerful tool to address this issue, since it allows researchers to follow the evolutionary process in real time (Kawecki et al. 2012; Magalhães and Matos 2012) and Acari are excellent models for such studies (Belliure et al. 2010). In particular, experimental evolution could be used to test the effect of contrasting environments on the development of reproductive isolation among populations. Curiously however, only a single study has thus far tested the effect of a contrasting evolutionary history upon the evolution of reproductive isolation, with negative results (Magalhães et al. 2009).

### Asexuality

While the role of sexual reproduction is well known in the process of speciation, asexual organisms (including some mites) may speciate in the absence of sexual recombination (e.g. Barraclough et al. 2003; Maraun et al. 2004; Birky et al. 2005; Fontaneto et al. 2007, 2008, 2009). Schwander and Crespi (2009) and Fontaneto et al. (2009) both suggested that strictly parthenogenetic reproduction and asexuality in general may promote cryptic diversity in microscopic animals. Data on cryptic mite species that are strictly parthenogenetic appear to be consistent with this idea. The oribatid mite *Platynothrus peltifer* is an ancient parthenogenetic species that has experienced high genetic diversification following patterns that are consistent with continental drift; such genetically divergent but morphologically similar lineages could be considered separate cryptic taxa (Heethoff et al. 2007). Speciation events have also been detected in another oribatid complex, *Tectocepheus* spp., in the absence of sexual reproduction (Laumann et al. 2007). Asexuality has arisen many times in the Acari, and many taxa reproduce entirely parthenogenetically (Walter and Proctor 2013). Given the potential for speciation and diversification in asexual lineages, the parthenogenetic Acari should not be neglected when exploring hidden diversity.

## Conclusions and future directions

The main conclusion of our analyses is that a symbiotic lifestyle in the Acari is not correlated with higher rates of cryptic speciation. Instead, we showed that cryptic species occurrence is closely related to the number of attempts made to detect them. Moreover, the use of both DNA tools and bioassays significantly increased the probability of distinguishing species not previously separated by morphological characters.

Cryptic species are common across all taxa and it is suggested that global animal and plant species diversity is grossly underestimated because of this (Bickford et al. 2007; Pfenninger and Schwenk 2007; Ceballos and Ehrlich 2009). In the case of microscopic animals, even less is known about the extent of their diversity. Many experts suspect the existence of significantly more cryptic diversity in Acari than is currently known (e.g. Walter and Campbell 2003; Knee et al. 2012; Stålstedt et al. 2013; Engelbrecht et al. 2014; Lareschi and Galliari 2014). On the basis of literature mining we show a demonstrable need for future investigations that will unveil potentially hidden mite species and identify evolutionary mechanisms behind cryptic speciation within Acari.

To improve detection of cryptic lineages, intensive sampling and application of molecular tools should be emphasized. Integrative approaches combining various methods (e.g. molecular, morphological, ecological and crossing) should be employed to effectively test for the presence of distinct Acari evolutionary lineages, while providing data to interpret their evolutionary histories. In numerous post hoc studies following separation of cryptic species using DNA tools, morphological key characters were discovered that supported the new species designations. In addition to traditional morphology and morphometry, new technologies, such as confocal laser scanning microscopy (CLSM) and low-temperature scanning electron microscopy (LT-SEM) have been employed in acarological studies (e.g. Valdecasas 2008; Chetverikov et al. 2013; Chetverikov 2014a, b; Rezende et al. 2015) and their more widespread availability would allow more detailed observations and thus distinctions between taxa previously considered as identical.

Acarologists cannot imagine a world without the incredible diversity of mites and ticks, these tiny creatures that contribute tremendously to the breadth of life on Earth and provide fascinating research subjects. Given that the diversity of the Acari can be expected to be much greater than recognized today, unveiling its cryptic component will surely stimulate great scientific and human interest.
